# Screening, brief intervention, and referral to treatment (SBIRT) for offenders: protocol for a pragmatic randomized trial

**DOI:** 10.1186/1940-0640-8-16

**Published:** 2013-10-23

**Authors:** Michael L Prendergast, Jerome J Cartier

**Affiliations:** 1 Integrated Substance Abuse Programs, Semel Institute, David Geffen School of Medicine, University of California, 11075 Santa Monica Blvd., Suite 100, Los Angeles, CA 90025, USA

**Keywords:** Screening, brief intervention, and referral to treatment (SBIRT), Offenders, Jail, Protocol, Pragmatic randomized trial

## Abstract

**Background:**

Although screening, brief intervention, and referral to treatment (SBIRT) is an evidence-based technique that, in some health-care settings, has been shown to cost-effectively reduce alcohol and drug use, research on the efficacy of SBIRT among criminal offender populations is limited. Such populations have a high prevalence of drug and alcohol use but limited access to intervention, and many are at risk for post-release relapse and recidivism. Thus, there exists a need for treatment options for drug-involved offenders of varying risk levels to reduce risky behaviors or enter treatment.

**Methods/design:**

This protocol describes an assessment of SBIRT feasibility and effectiveness in a criminal justice environment. Eight-hundred persons will be recruited from a large metropolitan jail, with the experimental group receiving an intervention depending on risk level and the control group receiving minimal intervention. The intervention will assess the risk level for drug and alcohol misuse by inmates, providing those at low or medium risk a brief intervention in the jail and referring those at high risk to community treatment following release. In addition, a brief treatment (eight-session) option will be available. Using data from a 12-month follow-up interview, the primary study outcomes are a reduction in drug and alcohol use, while secondary outcomes include participation in treatment, rearrest, quality of life, reduction in HIV risk behaviors, and costs of SBIRT.

**Expected value:**

Individual reductions in alcohol and drug use can have significant effects on public health and safety when observed over a large population at risk for substance-use problems. With wider dissemination statewide or nationwide, a relatively low-cost intervention such as SBIRT could offer demonstrated benefits in this population.

**Trial registration:**

Clinical Trials Government Identifier, NCT01683643.

## Introduction

Nearly all people with a history of drug use enter the criminal justice system at some time in their drug use career, frequently on a recurring basis. Drug use is closely associated with crime, and its prevalence among offenders is high [1-3]. Drug use, particularly when involving injection, also contributes to increased risk of HIV transmission [4]. Relapse to drug use tends to occur within the first few months of release from incarceration [5,6], highlighting the importance of providing intervention options at the pre-release or reentry phase of the offender’s incarceration.

While many offenders use drugs at levels that do not necessarily require treatment, they are still at risk of progressing to abuse or dependence or of engaging in unhealthy behavior. Interventions for offenders at low or moderate risk are largely lacking within the criminal justice system. One strategy to address this would be to provide early intervention to offenders using approaches that are appropriate to level of risk. Such an intervention would provide appropriate care earlier than would otherwise be the case, curtailing progression to higher risk levels and reducing risky behaviors. Offenders who are serving a jail sentence, particularly on drug charges, are at a “teachable moment” in which they may be amenable to an intervention designed to reduce their risk of relapse and re-arrest and improve other behaviors.

Screening, brief intervention, and referral to treatment (SBIRT) provides universal low-cost screening to a target population using brief, valid, and reliable screening instruments. Based on results from the screening, counselors, health educators, or other staff can identify people at different risk levels and provide types and intensities of intervention in accordance with the level of risk, ranging from information or brief intervention for low-risk users to referral to formal treatment for high-risk users [7]. Through a combination of prevention/early intervention and formal treatment, SBIRT is a public health approach intended to have a positive impact on the drug- and alcohol-related behavior of a broad user population, rather than on the much smaller population of those diagnosed with abuse or dependence. Studies in health-care settings have reported that the costs of SBIRT are relatively low, and that the benefit-cost ratio is favorable ($3-$4 for every dollar spent) [8].

Less research has been conducted on brief interventions or SBIRT for drugs than for alcohol. But many randomized studies of brief intervention for drug use have found statistically significant effects for at least one of the primary outcomes [9-15] an exception is [16]. Also, published articles on SBIRT projects for drug use funded by the US Substance Abuse and Mental Health Services Administration (SAMHSA) have reported significant reductions in drug use and other problems from baseline to follow-up [17-20], although use of a single-group design in these projects precludes strong conclusions about the causal effect of SBIRT on drug use. Also, limited research bearing on the use of brief intervention or SBIRT with offenders is available. Three randomized studies suggest that brief intervention can result in positive behavioral change among offenders [12,21,22].

In summary, although SBIRT has been found to be effective in some populations in health-care and other settings, it remains an empirical question whether SBIRT is a feasible intervention for offenders and whether it encourages treatment participation, reduces substance use, and results in other longer term benefits. Given the large proportion of offenders who use drugs and alcohol and who experience problems associated with such use, a relatively low-cost intervention such as SBIRT could have significant public health and public safety implications.

### Study aims

The aims of the SBIRT for Offenders study are to a) assess the effectiveness of SBIRT with jail inmates in terms of participation in a graduated series of interventions (brief intervention, brief treatment, and referral to longer term treatment), depending on risk level; b) determine the effectiveness of SBIRT with jail inmates on public health and public safety outcomes at 12 months following study admission, namely, drug use, alcohol use, criminal activity, arrest, reincarceration, HIV risk behaviors, and quality of life; and c) determine the cost of providing an SBIRT intervention to jail inmates. To accomplish these aims, we will recruit jail inmates who are completing their sentence in a large Los Angeles County jail. The study will be a collaboration between the University of California at Los Angeles (UCLA) Integrated Substance Abuse Programs (ISAP), the Los Angeles County Sheriff’s Department, Homeless Health Care Los Angeles (HHC-LA), and the Substance Abuse Prevention and Control Division of the Los Angeles County Department of Public Health.

## Methods/design

### Setting and participants

Screening and brief intervention will occur in two jails in Los Angeles County. Men’s Central Jail houses approximately 5000 male inmates from throughout Los Angeles County. The Twin Towers Detention Facility houses about 400 female inmates from throughout the county. The majority of inmates at both facilities have been sentenced on misdemeanor charges. As noted above, brief treatment will be conducted by trained counselors from HHC-LA, either in person at the HHC-LA treatment program or by telephone. Participants needing longer term treatment will be referred to one of the county’s Community Assessment Services Centers.

Since SBIRT is intended to be a universal intervention within a target setting, all adult (18+) male and female inmates who are within two weeks of scheduled release and who are available during the times that recruitment is conducted will be eligible to participate, with the following exceptions: those who 1) lack fluency in English or Spanish, 2) are unwilling to provide locator information for follow-up, 3) plan to leave the Los Angeles area within 12 months, 4) already have a referral to treatment following release from jail, or 5) are unable to provide informed consent owing to cognitive impairment.

### SBIRT for Offenders intervention

The conceptual framework of SBIRT is informed by empirical and theoretical considerations. There is a continuum of substance use problems and awareness of those problems among users. Screening, brief intervention, and referral to treatment systematically identifies, through screening, an individual’s level of risk and provides (or at least offers) an intervention that is appropriate to the assessed level of risk. The type and intensity of the intervention depend on the level of risk and the degree to which the individual is ready to make changes, as posited in the Stages of Change model (Pre-Contemplation, Contemplation, Preparation, Action, and Maintenance [23]). Most participants in the study will be in the Pre-Contemplation or Contemplation stage and, in any case, are not likely to be actively seeking treatment. For the brief intervention component of SBIRT, the therapeutic technique to help clients move to the Preparation or the Action stage is typically motivational interviewing, using the FRAMES model (Feedback, Responsibility, Advice to change, Menu of options, Empathy, and Self-efficacy [24]). The SBIRT model used in the study will include the components typical of SBIRT models generally: 1) screening to identify level of risk and, depending on the level of risk, 2) information, 3) brief intervention, and 4) referral to treatment.

### Screening and risk-level interventions

Screening is intended to identify individuals at varying levels of risk associated with the use of alcohol and/or drugs. It is not intended to provide a formal diagnosis (which is the function of clinical assessment), but rather to provide guidance for deciding which level of intervention may be needed to address the identified level of risk. As explained below, all participants in the study will complete the Alcohol, Smoking, and Substance Involvement Screening Test (ASSIST) [25] for screening. The ASSIST is an eight-item instrument developed by the World Health Organization (WHO) to screen for hazardous, harmful, and dependent use of alcohol, tobacco, and drugs. The ASSIST typically takes about 5-10 minutes to administer, although it can take longer depending on the number of drugs used over the lifetime. For each drug endorsed, questions are asked about frequency of use in the three months prior to the current incarceration, problems related to use, dependence indicators, and injection drug use. Following ASSIST administration, separate risk scores are calculated, with scores falling within a low-, moderate-, or high-risk range. Table 1 shows the risk-level scores for alcohol and drugs and the indicated intervention for each risk level, as specified in the WHO brief intervention manual [26]. We will use a computer-based version of the ASSIST to make the screening faster to administer and score.

**Table 1:** ASSIST risk scores and level of intervention

	**ASSIST alcohol score**	**ASSIST drug score**	**Intervention**
Low risk	0-10	0-3	Feedback on ASSIST score, literature
Moderate risk	11-26	4-26	Feedback on ASSIST score, literature, brief intervention
High risk	27+	27+	Feedback on ASSIST score, literature, brief intervention, referral to treatment

#### Low risk for drug and/or alcohol use

Study participants in the SBIRT group who score at low risk on the ASSIST for drug or alcohol use are notified by the health educator of their screening score and its meaning and are given literature on drug and alcohol use, HIV risk behaviors, and HIV testing. The health educator provides clients with a list of treatment resources should their behavior change in the future or to share with affected family or friends.

#### Moderate risk for drug and/or alcohol use

For users of drugs and alcohol who score at moderate risk on the ASSIST, the health educator provides feedback on the screening score and provides a brief intervention to encourage cessation of use, a description of brief treatment and its benefits, and a referral to brief or other treatment if the client requests it. The health educator also provides participants at this risk level with literature on the effects of drug and alcohol use and on HIV risk behaviors and HIV testing. (Although the ASSIST assesses tobacco risk, because of time constraints the intervention will not address tobacco use; however, the literature provided will include advice on smoking cessation).

The brief intervention takes about 15-20 minutes and uses a motivational interviewing approach. The health educator reviews the screening score and its meaning, assesses readiness to change, establishes goals with the client, and reviews strategies for change. One of the strategies is the opportunity to participate in a brief treatment intervention upon release from jail. If requested by the client, the health educator provides a referral to more intensive treatment.

Brief treatment (BT) is an individual-based intervention that is intended for clients who score at moderate risk. It is designed mainly to help clients who have few complicating problems to learn and develop skills to change their behavior. It is also appropriate for those who score at high risk but who are unwilling to commit to longer term treatment or for those who are on a waiting list for longer term treatment. It is offered at no cost to participants assigned to the SBIRT condition. The study will use a BT model developed for a SAMHSA-funded SBIRT program in San Diego, which was also used in a previous Los Angeles SBIRT project; it has been modified as needed for use in the study. This manualized treatment utilizes elements of two evidence-based practices: motivational interviewing and cognitive behavioral therapy [24,27,28] and consists of eight highly structured sessions delivered in person or by telephone. The initial session consists of orientation to BT and a needs assessment using the Addiction Severity Index-Lite (ASI-Lite) [29]. In Sessions 2-7, the counselor builds on the findings of the initial assessment by monitoring the client’s progress toward meeting self-defined goals in the areas of substance use, relapse prevention, HIV risk behaviors, and other identified psychosocial needs. In Session 8, the counselor reviews with the client his or her progress toward meeting self-defined goals, provides positive verbal reinforcement, and discusses future steps towards goals. If the client requests more intensive treatment, the counselor provides a referral as described below.

#### High risk for drug and/or alcohol use

For clients who score at high risk, the health educator gives feedback on the screening score and conducts a brief intervention to encourage the client to accept and follow through on a referral to a specialty treatment program. Information on HIV risk behaviors and testing is also provided. The health educator refers clients to one of 19 Community Assessment Services Center (CASC) offices located throughout Los Angeles County; the CASCs conduct assessments for substance use disorders and other problems and then refer clients to appropriate treatment programs, most of which offer comprehensive services, including HIV/AIDS education and HIV testing and counseling. The health educator provides the client with the name, telephone number, and address of the CASC located near where he or she lives or works. The county Department of Public Health has multiple sources of funding for persons who wish to receive treatment but do not have health insurance. Should the client prefer BT, the health educator sends the person’s contact information to the counselor, who will arrange for an in-person or telephone appointment.

### Selection and training of SBIRT health educators and counselors

Homeless Health Care-Los Angeles (HHC-LA) will provide SBIRT to jail inmates assigned to the intervention group. The HHC-LA health educators (who provide screening and brief intervention) and counselors (who provide BT) will be individuals who have experience working with offenders with substance use problems. They will need to receive clearance to work in the jails. They will participate in a week-long training in study design, administration, and scoring of the ASSIST, brief intervention, BT, and procedures for referral to treatment. Training will be provided by the principal investigator (study purpose and design), the project director (ASSIST, brief intervention, referral), and a consultant on BT. Training in the components of the intervention will be conducted in a small-group format with use of presentations, demonstrations, role-playing, and videotaping of practice sessions to ensure that the health educators and counselors are familiar with the material, have mastered the techniques, and have received appropriate feedback. The BT consultant will provide refresher training every six months. In addition, a clinical supervisor at HHC-LA will hold weekly meetings with health educators and counselors. Staff will be bilingual in English and Spanish, and literature will be available in English and Spanish.

### Human subjects approval

The study procedures and the informed consent form have been approved by the UCLA General Campus Institutional Review Board.

### Power analysis for sample size

Average effect sizes from meta-analyses of brief interventions for alcohol problems [30-32] range from a standardized mean difference of 0.18 to 0.43. Although no meta-analyses have been conducted on brief interventions for drug problems, effect sizes from primary studies involving illicit drugs [9-11,14,33] range from 0.13 to 0.84. Most of these studies focus on clients in primary care or other health-care settings. No experimental studies have reported outcomes for SBIRT (as opposed to brief intervention) with offenders. Since previous research provides limited guidance for estimating the expected effect sizes for the primary outcomes of the study, it seems reasonable to assume a small effect size; that is, a standardized mean difference of about 0.20 for a reduction in alcohol and drug use from baseline to 12-month follow-up. The power analysis (using the RMASS2 program; [34]) assumes alpha = 0.05 (two-sided), power = 0.80, an attrition rate of 15%, and an autoregressive variance-covariance structure, with a moderate correlation of 0.50 between levels of use at baseline and follow-up. With an expected effect size of 0.20 for the primary outcomes, the sample size is 778, equally divided between the two study groups. This estimate is similar for different variance-covariance structures, including a random-effects structure. We set the total sample at 800. To assess possible treatment by gender interactions, the sample recruited will include 25% women.

### Recruitment, randomization, and baseline interviews

We expect to recruit approximately 33 participants per month, although recruitment over the first three or four months may be somewhat lower given the time that it will take to establish a smooth and efficient process for recruitment and intervention within the jails. Recruitment should take about 24 months. Based on previous studies of brief intervention or SBIRT that reported refusal rates, we estimate that about 25% of inmates approached will refuse to participate.

Each week, Los Angeles County Sheriff’s Department staff will provide ISAP research staff with a list of names, identification numbers, and scheduled release dates for inmates who are eligible for study participation for that week. On designated recruitment days, research staff will request to see inmates prioritized by release date. Custody staff will call up inmates and escort them to the recruitment location within the jail.

The interviewer will explain the nature of the study and review the informed consent form with the potential participants. If the person agrees to participate, the researcher will obtain the required signatures on the consent form and provide a copy to the participant. Using a set of randomly ordered opaque envelopes prepared by ISAP’s Data Management Center, the interviewer will randomly assign the participant to the SBIRT group or the comparison group and will inform him or her of the assignment. For inmates who refuse to participate in the study, the interviewer will record the reason(s) provided, if any, and basic gender, age, and race/ethnicity based on visual cues.

If the participant is randomized to the SBIRT group, the interviewer will administer the baseline interview, obtain locator information for the follow-up interview, and then direct the person to the health educator, who will administer the ASSIST; the participant will then receive the appropriate intervention based on ASSIST score.

If the participant is randomized to the control group, the interviewer will administer the baseline interview and locator form and then administer the ASSIST. Having the interviewer administer the ASSIST to the control participants accomplishes two purposes: first, it ensures that we have comparable data on risk levels for participants in both study groups, and second, it ensures that the control participants receive the ASSIST in a research context rather than in a clinical context, thus strengthening the clinical contrast between the two groups. (As discussed later, there may still be a research effect on outcomes; however, this is unavoidable if we wish to collect self-report baseline data that cannot be obtained from records.) Following the interview, the interviewer will inform control participants of their risk level based on the ASSIST and will provide them with literature on reducing drug and alcohol use and HIV risk behaviors and a list of treatment programs in Los Angeles County. Control participants will not receive a brief intervention or a referral to treatment, but they may, of course, decide to enroll in treatment or seek other help on their own, which will be determined in the follow-up interview or through records.

### Data sources

Research staff will be bilingual in English and Spanish, and consent forms, instruments, and other materials will be available in English and Spanish.

#### Baseline interview

Given the setting, the nature of the intervention, and the need to encourage participation, the baseline interview will be kept as brief as possible (about 30 minutes, rather than the hour or more that baseline interviews usually take). The domains covered will include demographics, drug and alcohol use, treatment history, stage of change and readiness for treatment, HIV risk behaviors, and crime and criminal justice history. Drug and alcohol history will be available from the ASSIST and other standard drug-use assessments. Other items will be drawn from the Intake Instrument of the Criminal Justice Drug Abuse Treatment Studies Cooperative (funded by the National Institute on Drug Abuse [NIDA]); the University of Rhode Island Change Assessment Scale (URICA [35]); and the HIV risk-behavior questions used in the NIDA Clinical Trials Network.

#### Follow-up interview

The 12-month follow-up interview will last about one hour and will include questions and scales that measure outcomes, namely, drug and alcohol use (using the ASSIST), participation in treatment, quality of life, HIV risk behaviors, crimes committed, and criminal justice contacts. Quality of life will be measured with the WHO Quality of Life–Bref Survey [36]. All participants (SBIRT and control) who report having received treatment since baseline will also be asked about their satisfaction with the treatment they received (Client Satisfaction Questionnaire-8 [37]).

#### Biological measures for drug and alcohol use

At the follow-up interview, participants will be requested to provide two oral fluids samples, one of which will be analyzed for drugs, the other for alcohol, both using onsite test kits [38,39]. Compared with urine testing, oral fluid testing is less intrusive, is more convenient, is more easily observed, makes adulteration more difficult, and provides results that have sufficient sensitivity and specificity for research purposes. The drug test will provide results for opiates, cocaine, methamphetamine, amphetamine, marijuana, and phencyclidine—the drugs most commonly used in southern California. At the direction of our Institutional Review Board, we will not collect oral fluids at follow-up interviews conducted with incarcerated subjects. In addition, oral fluids samples will not be collected from participants who are interviewed by telephone.

#### Administrative records

We will obtain records-based data on arrests and incarceration over the follow-up period from the Los Angeles Sheriff’s Department and the California Department of Justice and on participation in publicly funded treatment from the Substance Abuse Prevention and Control office of the Los Angeles County Department of Public Health.

#### Cost data

Data on the costs of the SBIRT intervention will be collected using the Brief Drug Abuse Treatment Cost Analysis Program (http://www.datcap.com[40]), which has been used to conduct cost analyses of a variety of drug abuse treatment programs, including brief intervention [41]. Costs will be collected in Year 3 and will include staff training and monitoring; staff time for screening, brief intervention, and BT; time spent by sheriff’s department employees; equipment and supplies; and other costs associated with the intervention. Given the lack of research on the use of SBIRT with offenders, it is premature to conduct a full cost-effectiveness or benefit-cost analysis. However, the cost analysis will provide policy-relevant information with regard to the cost of providing SBIRT to a jail population. Should the intervention prove to be effective in this study, the cost data will lay the foundation for a proposal to conduct a comprehensive benefit-cost analysis of SBIRT with offenders.

### Interview training and procedures

Prior to training, interviewers will need to receive clearance to work in the jails. The ISAP Training Unit and project research staff will provide a five-day training that consists of the regular ISAP training modules required of all interviewers: Good Research Practices, Safety Concerns in Dealing with Patients, and Data Collection Procedures. The training will also cover 1) familiarization with study aims, instruments, and interview procedures specific to the study; 2) confidentiality and informed consent, data integrity, and data security; 3) issues and cautions in working with offenders; 4) observation of mock interviews conducted by the project director; and 5) practice interviews with the project director. Interviewers will also receive “red flag” training on how to recognize and respond to danger to self and others, child abuse, elder/dependent adult abuse, and domestic violence. Interviewers will receive annual refresher training on these topics.

The baseline interviews will be conducted in a private office at the jail. The follow-up interviews will occur at locations that are safe, ensure privacy, and minimize travel costs. Follow-up interviews may be conducted in jails or prisons. If a face-to-face interview is not possible (e.g., the participant is located out of state), the interview will be conducted by telephone. When setting up the follow-up interview, and again at the interview, the interviewer will confirm the identity of the participant by asking for full name and birth date. As a quality check, the project director will call a 10% random sample of participants for whom follow-up interview forms are on file to verify that the interview took place. Weekly project meetings with research staff will enable problems in recruitment and data collection to be quickly identified and addressed. Several procedures will help standardize data collection and encourage accuracy and honesty in reporting, as follows: 1) trained interviewers will conduct the interviews; 2) a private interview setting will minimize distractions and ensure confidentiality; 3) interviewers will remind participants of the confidentially of the information that they provide; 4) interviewers will be attentive to signs of fatigue and offer breaks if necessary; and 5) interviewers will be trained to look for reporting inconsistencies and socially desirable versus honest responses.

Participants will be paid $20 for the baseline interview, $50 for the follow-up interview, and $10 for two voluntary oral fluids samples (one for drugs, the other for alcohol) at follow-up. For interviews in the community, payment will be in the form of a gift card provided immediately following the interview (or mailed if a telephone interview). For interviews conducted in jail or prison, payment will be in the form of either money order or cash (depending on institution procedures) deposited to the inmate’s trust account.

### Tracking and locating for 12-month follow-up interviews

At baseline, the interviewer will ask participants to fill out a Locator Form. This form contains information that is useful for locating participants for follow-up interviews (e.g., driver’s license; names, addresses, and phone numbers of immediate relatives and unrelated friends; areas of town frequented; locations where social services are received; and probation or parole officer contacts). Other tracking resources include local and state correctional agencies that maintain publicly available websites that can be searched to determine whether a subject is in jail or prison or is on probation or parole. We will use a program developed in FileMakerPro^®^ for previous ISAP studies to record information needed for the tracking and locating process (e.g., locator data, agency contacts, letters, telephone calls, appointments). Tracking and locating procedures are described in a manual that was developed at ISAP [42] and is widely distributed by NIDA and CSAT. These procedures have been used by ISAP researchers in many studies to track and locate subjects for as long as 10 years between interview points, with follow-up rates typically at 85% or more [43]. Follow-up interviews have been conducted with subjects who are homeless, who are in jail or prison, who have absconded from probation or parole supervision, or who reside in other states or countries. In short, we expect that the detailed locator information from participants and the successful strategies used in previous studies will enable us to locate 90% of the baseline sample and, allowing for refusals and deaths, to interview at least 85% of the sample at the 12-month follow-up. We will obtain criminal justice and treatment records covering the 12-month follow-up period on all participants.

### Analysis plan

#### Variables

The independent variable for analysis is treatment status (i.e., assignment to either the SBIRT group or the comparison group). With respect to dependent variables, in accordance with Consolidated Standards of Reporting Trials (CONSORT) recommendations for randomized trials [44], we divided the dependent variables (outcomes) into primary and secondary. The power analysis is based on detecting effects for the primary outcomes. The two primary outcomes are reductions in the use of drugs and of alcohol (based on self-report and biological testing). The secondary outcomes are participation in treatment (based on self-report and records) and in self-help groups (based on self-report); rearrest and incarceration (based on records); quality of life (based on self-report); and HIV risk behaviors (based on self-report).

#### Hypotheses

The analyses will test the two primary hypotheses and four secondary hypotheses. The primary hypotheses are that, over the 12-month follow-up period, clients in the SBIRT group will be 1) more likely to reduce their level of drug use and 2) more likely to reduce their level of alcohol use compared with clients in the control group. The secondary hypotheses are that, over the 12-month follow-up period, clients in the SBIRT group will be 1) more likely to participate in brief treatment and/or longer-term treatment and to participate in AA, NA, or other self-help groups; 2) less likely to be arrested and incarcerated; 3) more likely to have a higher quality of life; and 4) less likely to engage in HIV risk behaviors compared with clients in the comparison group.

#### Research questions

The study will also examine several research questions related to participation, implementation, and cost:

Participation

1. How many inmates agreed to be in the study? What reasons were given for any refusals?

2. Of those assigned to the SBIRT group, what percentage was screened?

3. Of those screened, what percentage scored at each risk level?

4. Of those who scored at moderate or high risk, what percentage participated in brief treatment and/or longer-term treatment?

5. What was the level of satisfaction with the type of intervention received?

Implementation

6. What problems were encountered in setting up and implementing SBIRT in a jail setting?

7. What problems were encountered in recruiting participants and in delivering the intervention?

Cost

8. How much does it cost to provide the SBIRT intervention to men and women inmates?

#### Preliminary analysis

Equivalency between study groups will be examined on baseline characteristics that are correlated with outcomes. Attrition bias will be examined using baseline characteristics to compare those who complete follow-up with those who do not. Oral-fluid test results will be used to assess the validity of self-report of drugs and alcohol. Records data will be compared with self-report on treatment participation and criminal justice involvement.

#### Missing data

The main strategy to reduce missing data will be to train and monitor interviewers to ensure that all questions are answered. In analyses, missing data will be explored through frequency distributions. Missing data will be addressed using maximum likelihood (ML) or multiple imputation (MI) estimates for incomplete data [45,46]. Alternatively, statistical techniques that allow for missing data may be used (e.g., generalized linear models).

#### Hypothesis testing

Data from the baseline and the 12-month follow-up interviews and from records will be used to test the hypotheses. Analyses will be based on the intent-to-treat principle. Primary outcome 1 for drug use and primary outcome 2 for alcohol use, measured as dichotomous outcomes at follow-up, will be tested using chi-square analysis. Measured as change over time, the two hypotheses will be tested using repeated measures Generalized Estimating Equations (GEE) modeling, available in SAS proc GENMOD [47]. If the two groups differ significantly at baseline on drug or alcohol use variables or on other variables, logistic regression analysis will be used to assess the group effect for the drug and alcohol use outcomes, controlling for the covariates. Secondary outcome 1 for participation in treatment and self-help groups will be measured as a dichotomous variable and will be analyzed using either chi-square or logistic regression analysis. For secondary outcome 2 on arrest and incarceration, Cox proportional hazards regression [48] will be used to analyze the group difference in terms of elapsed time to re-arrest and re-incarceration. For secondary outcome 3, quality of life, the score on the WHO Quality of Life measure is continuous and will be analyzed using t-tests or, if baseline covariates need to be controlled, a multiple regression model. The outcome for secondary outcome 4, HIV risk behavior, will be measured as involvement in total number of risky behaviors and will be analyzed using a *t*-test or, if there is a need to control for baseline variables, by using a multiple regression model.

For the primary outcomes, an effect size will be calculated. The index of effect size will be the standardized mean difference (calculated as the difference between the means on the outcome variable for the two groups divided by the pooled standard deviation); for proportions, the equivalent effect size is calculated using the arc sine transformation [49]. Effect sizes will provide a quantitative measure of the magnitude of the difference between groups; they will also be useful in calculating power for future studies of SBIRT.

#### Cost analysis

Cost analysis will focus on estimating and comparing total intervention costs for the SBIRT and the comparison groups. This will allow us to report both the total and incremental cost of SBIRT. The incremental cost is the additional cost (above the cost of services offered to comparison clients) of SBIRT. Specifically, we will calculate total annual cost, average weekly cost per client, and average cost per treatment episode for both groups. To highlight the relative contribution of the cost components, we will calculate the percentage of total annual cost accounted for by each cost category. The data analysis routines for the DATCAP have been used in prior studies [50,51].

#### Other analyses

Additional analyses, while not testing specific hypotheses, will address related topics of interest, such as differences in outcomes by risk level, stage of change, and readiness for treatment. Since such analyses may involve subgroups with insufficient cell size to meet power requirements, findings will be considered tentative.

## Discussion

The purpose, setting, and design of the study of SBIRT with jail inmates make it a pragmatic trial, as defined by Zwarenstein et al. [52], namely, a study with design choices that “maximise applicability of the trial’s results to usual care settings, rely on unarguably important outcomes such as mortality and severe morbidity, and are tested in a wide range of participants.” The study is expected to advance knowledge of SBIRT in several ways. First, virtually all of the evaluation and dissemination work on SBIRT has occurred in health-care settings, with little or no attention to the potential benefit of SBIRT for the large population of offenders, most of whom are at risk for drug and/or alcohol problems and for rearrest or supervision violation. Although a few studies have evaluated brief interventions with offenders, this study will examine the effectiveness of the full spectrum of SBIRT components. To our knowledge, this would be the first experimental study of the effectiveness of SBIRT with offenders. Second, whereas most previous research on SBIRT has focused on alcohol, the study will expand the relatively limited evidence base on the effectiveness of SBIRT with persons who use drugs (or drugs and alcohol). Third, the study supplements the brief intervention and the treatment referral components of SBIRT with a BT protocol for offenders who are at moderate risk or for those who are not willing to commit to longer-term treatment. Fourth, unlike most studies of SBIRT, this study will examine the effects of SBIRT on HIV risk behaviors. Finally, the study will collect needed information on the costs associated with providing SBIRT to an offender population. The study may face a number of challenges that may limit the validity or generalizability of the findings, although we will take steps to limit their effect.

### Recruitment

Given the large number of inmates being released from the jail each day, recruitment goals should be able to be met even with a relatively high refusal rate. But if numbers do fall short, we will identify reasons that inmates refuse participation and attempt to correct them, confer with jail staff to address any logistical issues, extend the recruitment period, or recruit at other county jail facilities.

### Randomization violations

The project director will closely monitor the randomization protocol to ensure the integrity of assignment (i.e., there are no crossovers from one group to another). Should problems occur, solutions will be discussed with research and counseling staff. Even if crossovers do occur, analyses of hypotheses will use the intent-to-treat principle.

### Attrition and self-report data

Every effort will be made to retain all participants in the study and to interview all participants at follow-up. Tracking and locating procedures described above should minimize attrition. Virtually complete data will be available for outcomes based on administrative records. Although there is often concern about the validity of self-report, prior research indicates that self-report interviews, when properly conducted, are generally reliable and valid in measuring drug and alcohol use [53,54] and criminal involvement [55]. The strategies discussed above will increase reliability and validity of self-report data.

### Research effects

A number of researchers [10,56-58] have noted that the intervention effects of brief interventions may be confounded with research effects—that is, research assessments may have a therapeutic effect for all subjects, thus diluting treatment effects. Although this study will not formally assess this possibility through manipulation, we do attempt to reduce the impact of research effects by limiting the amount of data collected at baseline.

### Generalizability

The study participants will probably not be typical of inmates who would be the intended population of SBIRT in routine practice. They are willing to participate in research activities, are compensated for their participation in research, and receive an intervention from carefully trained and monitored counselors. These necessary research conditions would not be present in a typical setting that provided SBIRT to offenders, and thus, the effects found may be attenuated in a “real world” setting.

## Conclusion

Screening, brief intervention, and referral to treatment provides a bridge between primary prevention and treatment by conducting early identification and intervention with people whose current use may be low but who are at risk for future alcohol and drug problems and by referring those with likely abuse or dependence to longer-term treatment. The public health assumption informing SBIRT is that individual reductions in alcohol and drug use, when aggregated over a large at-risk population, can have significant health, public health, and public safety effects [59]. As noted above, while SBIRT has been found to be effective in some health-care settings (and to a lesser extent in colleges and universities), little published research is available on the use of SBIRT to identify offenders at different risk levels and to provide appropriate levels of intervention. Should SBIRT prove effective with this population, it would offer a proactive approach to help offenders reduce drug and alcohol use, HIV risk behaviors, and criminal behavior and improve their psychosocial functioning. As such, the wider dissemination of SBIRT to criminal justice settings statewide or nationwide could have a significant impact on public health and public safety.

## Competing interests

The authors declare that they have no competing interests.

## Authors’ contributions

MP developed the conceptual framework and design for the study and wrote the draft of the manuscript. JC was primarily responsible for developing the SBIRT intervention for offenders and assisted in the draft of the manuscript. Both authors read and approved the final manuscript.
